# Is action understanding an automatic process? Both cognitive and perceptual processing are required for the identification of actions and intentions

**DOI:** 10.1177/17470218221078019

**Published:** 2022-02-18

**Authors:** Emma L Thompson, Emily L Long, Geoffrey Bird, Caroline Catmur

**Affiliations:** 1 Department of Psychology, Institute of Psychiatry, Psychology & Neuroscience, King’s College London, London, UK; 2 Department of Clinical and Health Psychology, University of Edinburgh, Edinburgh, UK; 3 Department of Experimental Psychology, University of Oxford, Oxford, UK; 4 MRC Social, Genetic & Developmental Psychiatry Centre, Institute of Psychiatry, Psychology & Neuroscience, King’s College London, London, UK

**Keywords:** Action understanding, dual task, cognitive processing, mirror neurons, perceptual processing

## Abstract

The ability to identify others’ actions and intentions, “action understanding,” is crucial for successful social interaction. Under direct accounts, action understanding takes place without the involvement of inferential processes, a claim that has yet to be tested using behavioural measures. Using a dual-task paradigm, the present study aimed to establish whether the identification of others’ actions and intentions depends on automatic or inferential processing, by manipulating working memory load during performance of a task designed to target the identification of actions and intentions. Experiment 1 tested a novel action understanding task targeting action identification and intention identification. This task was then combined with two working memory manipulations (cognitive: Experiment 2; perceptual: Experiment 3) to determine whether action identification and intention identification are disrupted by concurrent cognitive or perceptual load. Both action identification and intention identification were impaired by concurrent cognitive and perceptual processing, indicating that action understanding requires additional perceptual and cognitive resources. These findings contradict a direct account of action understanding.

Accurate understanding of other people’s behaviour is crucial for successful social interaction (Marchant & Frith, 2009). To interpret others’ current behaviour and predict their future behaviour, we need to identify their actions and infer the intentions underlying those actions (Hamilton & Marsh, 2013). Two alternative neurocognitive processes have been proposed as the basis for this ability.

One influential account suggests that the ability to identify others’ actions and intentions is performed by mirror neurons (Rizzolatti et al., 1996; Rizzolatti & Fadiga, 1998). Mirror neurons, originally found in frontal area F5 of the macaque monkey, are cells which fire to both the observation and execution of actions (di Pellegrino et al., 1992). While direct recording of mirror neurons in the human brain is rare (but see Mukamel et al., 2010), several indirect studies have found evidence for neurons with “mirror” properties in areas such as the inferior frontal gyrus, ventral premotor cortex, and inferior parietal lobule (see Molenberghs, Cunnington and Mattingley, 2012, for a review). The firing of these cells, to both the observation and execution of actions, suggests that an observed action is mapped onto the same motor programme that is used to execute that action in the observer. The discovery of this pattern of neural responses led to the claim that such responses allow the observer both to identify the other’s action (Gallese et al., 1996) and to infer their underlying intention (Fogassi et al., 2005).

An alternative account suggests that identifying others’ actions and intentions is an inferential process, which may utilise information from perceptual and/or sensorimotor systems, including mirror neuron brain areas, but that this process is not performed by those systems alone (de Lange et al., 2008; Hamilton & Marsh, 2013; Spunt et al., 2011).

One way in which these two accounts can be distinguished is by their predictions regarding the involvement of direct (automatic) versus inferential (controlled) processes in the identification of others’ actions and intentions. Direct perception accounts specify that action understanding takes place without the involvement of inferential processes (Gallagher, 2008; Rizzolatti & Sinigaglia, 2007). For example, Gallagher (2008, p. 537) states, “The relevant contrast is . . . between perception and something added to perception, e.g., an inference or interpretation that goes beyond what is perceived,” but this claim that action understanding does not involve inference has yet to be tested empirically using behavioural measures. A well-established way of distinguishing automatic from controlled processes is to test the impact of adding a secondary task, such as one designed to load working memory (Baddeley, 2012; Baddeley & Hitch, 1974; Baddeley & Logie, 1999). Automatic processes, which by definition do not depend on cognitive control/executive function, should remain unaffected under high working memory load, whereas inferential processes should be disrupted.

As noted above, the ability to identify others’ actions and intentions has been termed action understanding (Rizzolatti et al., 1996; Rizzolatti & Fadiga, 1998). However, there is as yet no clear consensus on the definition of the term action understanding (Gallese et al., 1996; Kilner, 2011; Rizzolatti & Craighero, 2004; Rizzolatti & Sinigaglia, 2010; Spunt & Adolphs, 2014; Umiltà et al., 2001; see Cook et al., 2014; Hickok, 2009, for further discussion of this issue). This term may refer to at least two processes: action identification (determining the identity of an observed action based on the relative configuration of body parts involved in the action), or goal/intention identification (generalising across differences in body part configuration to extract the goal or intention underlying an observed action; Thompson et al., 2019). In the present study, we targeted both of these putative action understanding processes.

The aim of the present study was therefore to establish whether the identification of others’ actions and intentions depends on automatic or inferential processing, by manipulating working memory load during performance of a task designed to target both action identification and intention identification. Experiment 1 tested a novel action understanding task targeting these two processes. This task was then combined with two working memory manipulations using a dual-task procedure (Baddeley & Hitch, 1974) to determine whether action identification and intention identification occur automatically or whether, instead, these processes are disrupted by concurrent numerical (cognitive: Experiment 2) or visual (perceptual: Experiment 3) working memory load.

## Experiment 1

Experiment 1 sought to develop a new action understanding task, targeting action identification and intention identification.

One task design used in several functional magnetic resonance imaging (fMRI) studies to distinguish different action understanding processes involves two conditions: one targeting “how” an action is performed (i.e., action identification) and the other “why” the action is performed (i.e., intention identification; Spunt & Adolphs, 2014; Spunt et al., 2010; Spunt & Lieberman, 2012, 2013). However, a recent study claimed that the distinction between these conditions is confounded by level of abstraction (Spunt et al., 2016), with the stimuli presented in “how” conditions being lower in abstraction than those in the “why” conditions. Spunt et al. (2016) demonstrated that this confound explained the differences in activation of brain regions that had previously been attributed to the distinction between the “how” and “why” conditions.

Furthermore, the stimuli used in the how/why task included objects, the importance of which was not matched across the “how” and “why” conditions. For example, one block of trials in the “why” condition involved identifying whether the intention behind an observed action was “to be healthy,” for which hands acting on objects such as salad and beer were shown. For these trials, observation of the object alone, without attention to the hand actions, was sufficient to determine the correct answer. This condition may therefore be reflecting object processing ability, rather than specifically targeting action understanding.

The aim of Experiment 1 was therefore to develop and test a new action understanding task that targeted both action identification and intention identification while controlling for both level of abstraction and object processing.

### Method

#### Open science statement

We report for all experiments how we determined our sample size, all data exclusions, all manipulations, and all measures in the study. The experiments were not preregistered. Data for all studies are available at https://doi.org/10.18742/16930846.

#### Participants

The required sample size for Experiment 1 was based on a small to medium effect size (*d* = 0.47) found in pilot data between the response times (RTs) for the action and intention conditions in the action understanding task (see Supplementary Materials). Using G* Power 3 (Faul et al., 2007), a *t*-test power analysis using the difference between two dependent means (matched pairs) with one tail, effect size of .47, alpha of .05, and power of .8 determined that 30 participants were required. Thirty-two native English speakers were therefore recruited via a local volunteer database and took part in the experiment. One participant was removed due to failure to follow instructions correctly. This resulted in 31 participants (7 males, 5 left-handed) aged 18–40 years (*M* = 22.06, *SD* = 5.58). All participants were compensated with a small fee or course credits for their time. Experimental procedures for all experiments were approved by the King’s College London Research Ethics Committee and were carried out in accordance with the Declaration of Helsinki.

#### Stimuli

The stimuli consisted of still images depicting pantomimed hand actions. These were selected and adapted from video clips created by Molenberghs, Hayward, et al. (2012). While video stimuli provide more enriched action information than still frames, they are problematic for RT measures because they introduce variability linked to the timing of the action: for example, different actions will differ in terms of the point in the video at which the action can be discriminated from other actions, and furthermore, this timepoint may differ between participants. If participants respond when the action or intention is identified, the amount of perceptual information presented may differ across participants. Alternatively, if responses are not permitted until the end of the video, the precise moment at which the action or intention is identified cannot be determined, which precludes the use of RT measures. For these reasons, each video was converted into a still image, depicting the typical body configuration for that action (as determined through piloting; see below). In total, 20 images were selected (16 were used in the main task; 4 in practice trials).

Each image was assigned two corresponding word phrases: one relating to the configuration of hand parts depicted in the image (action identification), and the other relating to the motivation underlying the depicted action (intention identification). Each phrase consisted of two to three words starting with the word “to,” for example, “to turn” (action) or “to open” (intention). Several rounds of piloting were conducted during which participants were required to rate how well they thought each phrase described the image on a scale of 1 = *does not describe picture at all* to 5 = *describes picture very well* and to indicate whether each word used was an action or intention, on a scale of 1 = *definitely action* to 4 = *definitely intention*. Images and word phrases were adjusted following piloting until matching was achieved (see below and Supplementary Materials).

#### Stimulus matching

The word phrases in the action and intention conditions were matched on a range of variables including number of words, *t*(15) = 1.46, *p* = .164; number of characters, *t*(15) = 0.24, *p* = .812; word frequency, as determined by per million words in the SUBTLEX database (van Heuven et al., 2014), *t*(15) = 0.80, *p* = .435; pilot participants’ ratings of descriptiveness, *t*(15) = 1.25, *p* = .230; and—crucially—abstractness ratings (Brysbaert et al., 2014), *t*(15) = 0.76, *p* = .457; see Table 1. Importantly, pilot participants’ ratings of the action word phrases were significantly lower (i.e., more like an action) than the intention word phrases, *t*(15) = 4.08, *p* = .001, *d* = 1.32, supporting the separation of the word phrases into discrete action and intention categories.

**Table 1.** Values for the controlled variables in each action understanding condition.

Variable	Action identification		Intention identification	
	*M*	*SD*	*M*	*SD*
Number of characters	7.25	1.57	7.13	1.31
Number of words	2.13	0.34	2.00	0.00
Word frequency	4.53	0.60	4.31	1.00
Descriptive value	3.81	0.74	3.60	0.70
Word abstractness score	3.91	0.32	3.77	0.58
Action-intention score	1.62	0.32	2.31	0.52

Once the phrases were finalised, each image was paired with alternative mismatching action and intention phrases. This resulted in 64 trials (16 images; 4 word phrases per image), divided equally across the two action understanding conditions (action, intention). Within each condition, each image was presented once with a matching phrase and once with a mismatching phrase.

#### Procedure

For all experiments, stimuli were presented and responses recorded using Psychopy 2 (Peirce, 2007) running on a Dell Latitude E7470 with a 14-inch LCD monitor (resolution, 1,920 × 1,080; refresh rate, 59 Hz).

**Figure 1.**
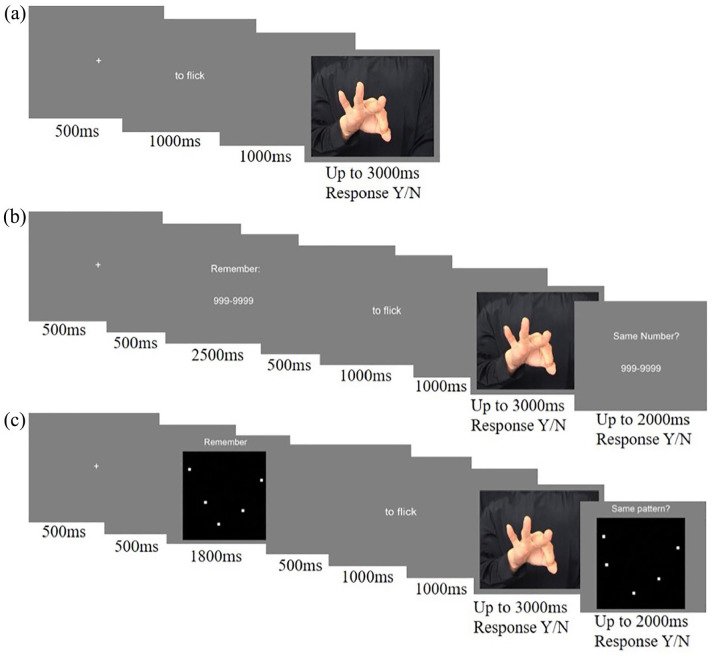
A single trial from each experiment. (a) Experiment 1 consisted of the action understanding task only. A trial from the action identification condition is illustrated here. (b) In Experiment 2, participants simultaneously completed the action understanding task and a cognitive load task (adapted from Spunt & Lieberman, 2013). An example of the low-load condition is illustrated here. (c) In Experiment 3, participants simultaneously completed the action understanding task and a perceptual load task (adapted from Mecklinger & Pfeifer, 1996). An example of the high-load condition is illustrated here.

Each trial commenced with a fixation cross (duration 1,000 ms) followed by a word phrase, which was presented on screen for 1,000 ms. After a blank screen for 1,000 ms, the corresponding image was presented until the participant responded, or for a maximum of 3,000 ms (see Figure 1a). Participants indicated with a yes/no response whether the word phrase described the image by pressing the x and m keys on the keyboard with their left and right index fingers, respectively. The mapping of response key (x or m) to response (yes or no) was counterbalanced across participants and was indicated to participants via green (yes) and red (no) stickers placed over the keys. Reminder stickers were also placed at the side of the screen. The 64 trials described above were presented in a random order twice; once in each of two blocks of trials. Participants were permitted to take a break for as long as required before completing the second block. Four practice trials (two action trials, one matching and one mismatching, and two intention trials, one matching and one mismatching) were presented in random order with feedback (“correct” or “incorrect,” presented on screen for 1,000 ms) before the first block. Data from these trials were not analysed.

Before the task began, participants were given written instructions and a familiarisation procedure was completed. Participants were told that they would see a word phrase on screen followed by an image of a hand action; that their task was to indicate whether the word phrase described the image, pressing the green button to indicate yes and the red button to indicate no. Participants were instructed to respond as quickly as possible, while maintaining accuracy. An example of the task was given: the experimenter suggested that the participant might see the phrase “to poke” followed by an image of a hand in a poking position, and the participant would press the green button to indicate this was correct.

Half the participants completed this task after completing another unrelated task, not relevant to the present study. The order of task completion had no significant effect on performance on the action understanding task, *F*(1,29) = 0.10, *p* = .755.

### Results

The RT data were filtered to remove all trials in which participants answered incorrectly. The mean (*M*) and standard deviation (*SD*) RTs for each condition (action and intention) were calculated for each participant, and outlying responses that were more than 2.5 *SD* from their corresponding mean were excluded. The proportion of correct responses and the mean RT were then calculated for each condition for each participant.

Inverse efficiency scores were calculated as a measure of overall performance on the task that would be suitable for analysis in the dual-task experiments (Experiments 2 and 3), as the current literature does not provide for a strong prediction for whether dual-task performance will impact on RT or accuracy, and in fact it is possible that different participants might use different strategies in terms of speed/accuracy trade-offs in a dual-task situation. RT and accuracy data are reported in the Supplementary Material for all experiments, along with multilevel model analyses of these data, the results of which are broadly consistent with those from the inverse efficiency analyses reported here.

**Figure 2.**
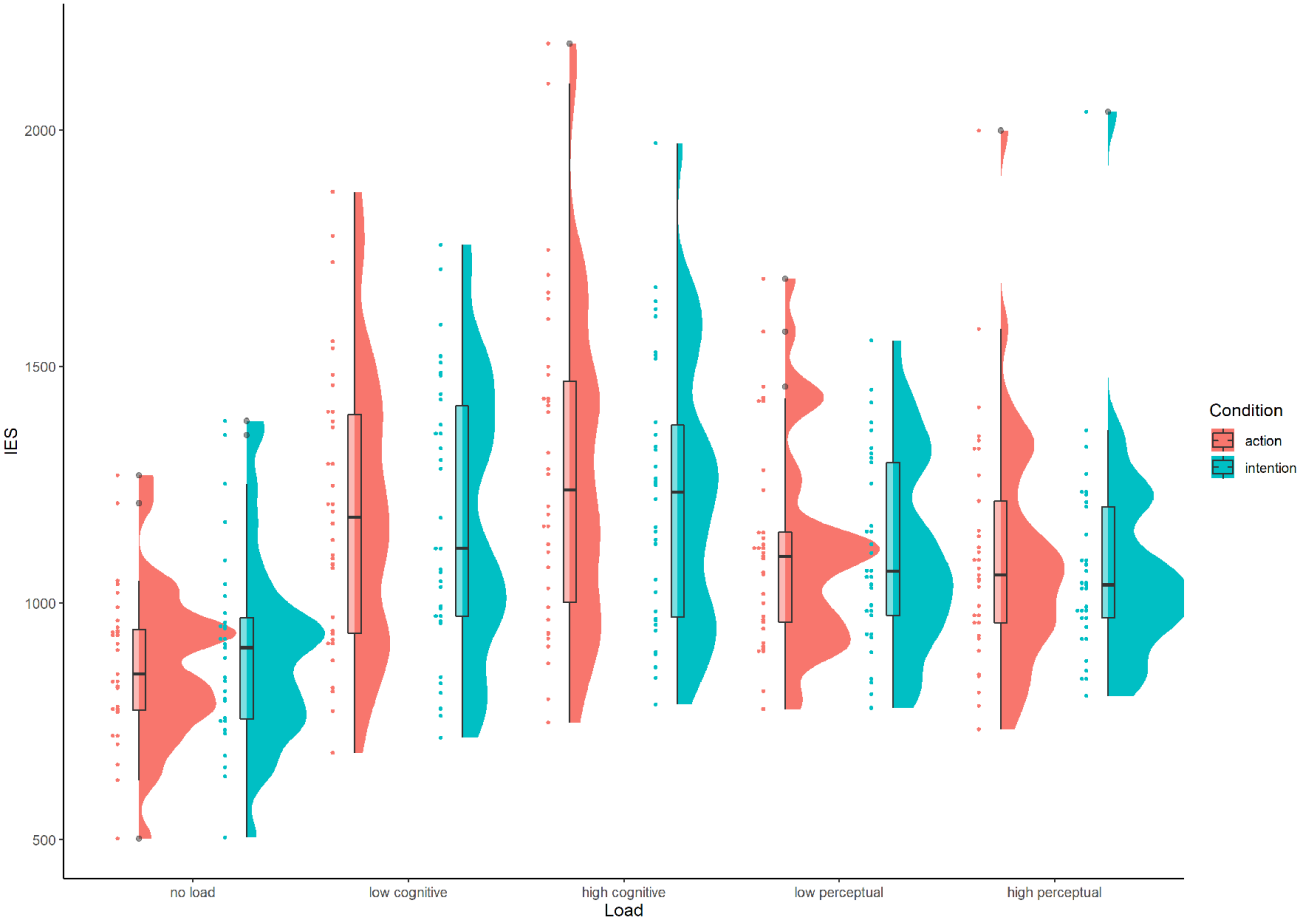
Inverse efficiency scores (IES) for each load and action understanding condition across the three experiments (Experiment 1: No load; Experiment 2: Cognitive load; Experiment 3: Perceptual load).

Inverse efficiency was calculated for each condition as RT/proportion correct responses, with higher scores indicating worse performance (slower and/or more errors, see Figure 2). A paired-sample *t*-test revealed a significant difference in inverse efficiency scores between the action (*M* = 869.2, *SD* = 161.3) and intention (*M* = 900.1, *SD* = 202.2) conditions, *t*(30) = 2.24, *p* = .033, *d* = 0.40.

### Discussion

The aim of Experiment 1 was to test a new action understanding task, targeting action identification and intention identification. The current task built on those used in previous studies (e.g., Molenberghs, Hayward, et al., 2012; Spunt & Adolphs, 2014; Spunt et al., 2016) and attempted to provide a more controlled measure of action understanding compared with tasks used previously. A range of variables were controlled across the action and intention conditions, including the level of abstractness, and stimulus confounds were also removed (e.g., by removing object processing demands). Any difference in performance between the two conditions can therefore be more confidently attributed to manipulation of the action understanding process (action or intention identification) than was possible in previous studies.

Participants demonstrated a significant difference in performance between the action identification and intention identification conditions, with worse performance on the intention identification condition. This difference indicates that when observing identical action stimuli, participants took longer and/or made more errors when asked to identify the intention underlying the observed action, than when asked to identify the action itself. This implies that action identification and intention identification may utilise different cognitive processes.

The significant difference in performance across conditions supports the results of Spunt and Adolphs (2014), whereby participants were significantly faster and more accurate in the how condition compared with the why condition. Although brain activation in that study may have been confounded by level of abstractness (Spunt et al., 2016), in Experiment 1 we controlled for abstractness. Our results therefore suggest that the performance difference between conditions in Spunt and Adolphs’ study may not have been due entirely to the abstractness confound but may instead be due to different cognitive processes occurring across the two conditions.

In Experiments 2 and 3, we used this task to measure action identification and intention identification performance during a dual-task procedure in which we also manipulated concurrent cognitive and perceptual working memory load.

## Experiment 2

Experiment 2 sought to determine whether action identification and intention identification depend on automatic cognitive processes. Automatic and controlled cognitive processes can be distinguished by the amount of attention and processing resources required (Posner & Snyder, 1975; Shiffrin & Schneider, 1977). Automatic processes occur without attention (Shiffrin & Schneider, 1977), awareness of initiation, or other general processing resources (Bargh, 1994). Controlled processes, however, require attention and are constrained by the amount of processing resource available (Shiffrin & Schneider, 1977). In regard to action understanding, if identifying actions and their underlying intentions are automatic processes, then they must be able to take place without attention and under limited processing resources. However, if action understanding requires additional inferential processing, performance of these processes will be impaired when attention and/or processing resources are unavailable. Experiment 2 therefore tested the automaticity of action and intention identification, by determining whether these processes are disrupted under limited working memory capacity.

Research investigating the impact of cognitive resources on task function typically uses a dual-task procedure (Baddeley & Hitch, 1974). This requires participants to complete the task of interest while simultaneously performing a secondary task that is known to load cognitive resources. The assumption underlying this method is that tasks which rely on the same cognitive processes will compete for cognitive resources when performed simultaneously, impacting on performance. Tasks that involve different processes will not compete, and as such performance will be unaffected. Consequently, dual-task costs only emerge when two tasks performed simultaneously involve the same cognitive processes.

In a previous fMRI study investigating the automaticity of responses in mirror neuron brain areas, Spunt and Lieberman (2013) adopted a dual-task design, using a concurrent working memory task to reduce cognitive capacity while participants completed an action understanding task. Participants were required to remember low-load numbers (e.g., 555–5555) or high-load numbers (e.g., 813–5467) while watching naturalistic videos of an actor performing an action. Participants completed one of four tasks while watching the video: observation only; understanding “what” the actor is doing; understanding “how” she/he is doing it; and understanding “why” she/he is doing it. The low-load numbers use minimal cognitive capacity, allowing for the processing of stimuli from the action understanding task. Therefore, brain responses to the action understanding task should not have been affected in the low-load condition. In contrast, the high-load numbers occupy the majority of cognitive capacity, leaving only automatic processing to be used for the action understanding task. If brain responses to the action understanding task were unaffected in the high-load condition, this would suggest that the processes involved are automatic, whereas reduced neural responses would indicate that controlled cognitive processes were required for the action understanding task.

Spunt and Lieberman (2013) found that responses in mirror neuron brain areas, specifically the ventral premotor cortex, left dorsal premotor cortex, and left anterior intraparietal sulcus, were unaffected by load regardless of the observer’s task. In contrast, when the observer was judging “why” the actor was performing the action, responses in mentalising brain areas, specifically the anterior temporal cortex and dorsomedial prefrontal cortex, were reduced under high cognitive load. These results suggest that mirror neuron brain areas may process others’ actions automatically, whereas mentalising areas are involved in inferring the intention underlying an observed action, and that this latter process requires greater cognitive resources (Spunt & Lieberman, 2013). However, as performance on the action understanding task was not recorded, Spunt and Lieberman’s study does not reveal whether there was a detrimental effect of increased cognitive load on task performance.

Experiment 2 therefore sought to measure action understanding performance under concurrent low- and high-cognitive-load conditions, by combining the action understanding task from Experiment 1 with a working memory task. This will determine whether action identification and/or intention identification are reduced under limited cognitive processing capacity.

Spunt and Lieberman (2013) found that responses in mentalising brain areas during intention identification (their “why” task) were reduced under high cognitive load. If these brain responses are related to performance on the intention identification task, then intention identification should be impaired under high cognitive load. However, Spunt and Lieberman found no reduction in brain responses to action identification (their “how” task) under high cognitive load, and therefore, based on their brain imaging data, action identification should not be affected by cognitive load (although, as behaviour was not measured in their study, a detrimental effect of cognitive load on action identification performance cannot be ruled out). At the purely behavioural level, if performance on either the action identification or intention identification tasks is reduced under high cognitive load, this would indicate that the affected task cannot take place automatically.

### Method

#### Participants

As no previous study has manipulated cognitive load during this task, we did not have an estimate of the specific effect size associated with this manipulation. As such we based our power analysis on the effect size of *d* = 0.40 found in Experiment 1, converted to an effect size of *f* = 0.205. Using G* Power 3 (Faul et al., 2007), an F-test power analysis conducted using an analysis of variance (ANOVA) repeated measures, within factors test, using an effect size *f* of 0.205, alpha level of .05, power of .8, 1 group, 4 measurements, 0.5 correlations between repeated measures, and nonsphericity correction of 1, resulted in a minimum sample size of 34. Based on this requirement, 35 native English speakers were recruited via King’s College London recruitment email. One participant was excluded as their performance on the action understanding task was not significantly greater than chance. This resulted in 34 participants (5 males, 1 left-handed) aged 19–60 years (*M* = 27.0, *SD* = 9.38). All participants were compensated a small fee for their time.

#### Stimuli

Stimuli for the working memory task were based on Spunt and Lieberman (2013) and comprised seven-digit number sequences. The low-load stimuli comprised seven identical digits, for example, 888–8888, while the high-load stimuli comprised seven different digits, for example, 813–5467. On each trial, participants were required to hold each sequence in working memory and subsequently (after the action understanding task) compare it with a comparison sequence. On half of trials, mismatching comparison sequences were presented. For these mismatching trials in the low-load condition, the stimuli comprised seven different identical digits, for example, 555–5555, while in the high-load condition, the position of four digits from the original stimuli were altered, for example, 831–6475. Digits were swapped systematically to ensure each position was altered an equal number of times while controlling for the frequency of each number in each position.

The number of working memory stimuli was matched with that of the action understanding stimuli, controlling for the same variables as in Spunt and Lieberman (2013): the frequency of each number; the frequency of each number in each position in the high-load conditions, for example, 3 being the third digit in the sequence; and the frequency of digits paired together in the mismatching low-load conditions, for example, 888–8888 with 555–5555. In total, there were 16 sets of stimuli in each of the following four sub-conditions: matching low-load, mismatching low-load, matching high-load, and mismatching high-load. A fully factorial combination of all action understanding stimuli and conditions with all working memory conditions was produced. This resulted in 256 trials, which were divided between four blocks of 64 trials per block, such that each block contained 16 trials per cell of the design (low-load action; low-load intention; high-load action; high-load intention), equally divided between matching and mismatching trials for both the action understanding and working memory tasks. However, piloting of the task indicated that four blocks was (a) too tiring for participants (taking 45 min to complete) and (b) encouraged the use of dual-task strategies (e.g., only remembering the first four numbers of the high-load sequences); thus, subsequently only two blocks of the task were utilised. These were randomly assigned to each participant.

The action understanding task was as described in Experiment 1.

#### Procedure

Participants completed the experiment in a single session. For each trial, participants completed the action understanding and working memory tasks simultaneously (see Figure 1b). Each trial commenced with a fixation cross (duration, 1,000 ms) followed by the working memory sequence (2,500 ms). A reminder “Remember:” was presented above the sequence, to avoid confusion between the encoding and answering phases. A blank screen (500 ms) was presented before and after the working memory stimulus. The action understanding task then began: a word phrase was presented (1,000 ms) followed by a blank screen (1,000 ms) and then the presentation of the image until the participant responded or for a maximum of 3,000 ms. Participants indicated with a yes/no response whether the word phrase described the image by pressing the x and m keys, with key-response mappings counterbalanced across participants as for Experiment 1. Finally, the working memory task was completed: the working memory comparison sequence was presented on the screen, below the reminder “Same?” for up to 2,000 ms. Using the same response keys and key-response mappings as for the action understanding task, participants indicated whether this number sequence was identical to the one at the beginning of the trial. In all, 128 trials were presented in a random order in two blocks of 64 trials. The trials in each block were equally distributed across the four conditions (low-load action; low-load intention; high-load action; high-load intention). Participants were permitted to take breaks for as long as required between blocks. Four practice trials (one per condition) were presented with feedback before the first block. Data from these trials were not analysed.

Before the task began, participants were given written instructions and a familiarisation procedure was completed. Participants were told that they were going to complete two tasks simultaneously; that the first task was a memory task, in which they had to remember a number sequence presented on screen; that while remembering the number, they would see a word phrase on screen followed by an image of a hand action; that their task was to indicate whether the word phrase described the image, pressing the green button to indicate yes and the red button to indicate no; that they would then see a second number sequence and their task was to indicate whether this was identical to the first one, again pressing the green or red button corresponding to yes and no, respectively. Participants were instructed to respond as quickly as possible, while maintaining accuracy, and to give equal importance to both tasks. As in Experiment 1, an example of the action understanding task was given.

### Results

The same RT filtering procedure was used as in Experiment 1. RTs from incorrect trials were removed. The mean and *SD* were then calculated for each working memory/action understanding condition: low-load action, low-load intention, high-load action, and high-load intention, with outlying responses that were more than 2.5 *SD*s from their corresponding mean excluded.

As in Experiment 1, inverse efficiency scores were calculated for each condition. For each participant, the mean RT for each condition was divided by the proportion of correct responses for that condition (see Figure 2). These calculations were also carried out for the responses on the working memory task (see Table 2).

**Table 2.** Inverse efficiency scores for each load and action understanding condition on the working memory tasks from both Experiments 2 (Cognitive) and 3 (Perceptual).

	Low load				High load			
	Action identification		Intention identification		Action identification		Intention identification	
	*M*	*SD*	*M*	*SD*	*M*	*SD*	*M*	*SD*
Cognitive task	823.4	237.0	811.5	209.1	1,346.3	334.3	1,303.3	300.7
Perceptual task	1,022.8	270.5	1,040.6	241.6	1,364.0	391.5	1,387.3	406.0

Inverse efficiency scores on the action understanding task were subjected to repeated measures ANOVA with within-subjects factors of cognitive load (low-load, high-load) and action understanding condition (action, intention). There was a significant main effect of cognitive load, *F*(1,33) = 11.26, *p* = .002, $\eta_{p}^{2}=25.4\%$ . Inverse efficiency scores were higher in the high-load (*M* = 1,256.5, *SD* = 317.8) than the low-load condition (*M* = 1,187.7, *SD* = 290.8). There was no main effect of action understanding condition and no interaction between load and condition.

Similarly, the inverse efficiency scores for the working memory task were subjected to the same repeated measures ANOVA. There was a significant main effect of cognitive load, *F*(1,33) = 220.19, *p* < .001, $\eta_{p}^{2}=87\%$ . Inverse efficiency scores were higher in the high-load (*M* = 1,324.8, *SD* = 316.3) than the low-load condition (*M* = 817.4, *SD* = 222.0), confirming that the high-load condition required greater cognitive resources, as intended. There was no main effect of action understanding condition and no interaction between load and condition.

### Discussion

The aim of Experiment 2 was to determine whether action identification and intention identification can take place automatically, or whether instead they require cognitive processing resources. Across both the action identification and intention identification conditions, participants performed significantly worse in the high-load condition compared with the low-load condition. This indicates that when cognitive capacity is limited, both action and intention identification are impaired, suggesting that both processes require controlled cognitive processing.

The finding that intention identification is impaired under limited cognitive capacity supports the findings of Spunt and Lieberman (2013), and suggests that the reduction in neural response observed in mentalising areas in their study during intention identification under high cognitive load may be linked to impaired performance on the intention identification task.

Intriguingly, action identification was also found to be impaired under limited cognitive processing resources. To some extent this contradicts the results of Spunt and Lieberman (2013), who did not find reduced responses in either mirror neuron or mentalising brain regions under limited cognitive processing resources in their “how” condition. However, the lack of a reduction in neural response does not necessarily imply that behavioural responses would have been intact, had they been measured in that study.

The results of Experiment 2 suggest that reducing cognitive processing resources had a detrimental effect on action and intention identification. Therefore, both action identification and intention identification require cognitive processing resources, indicating that neither process takes place automatically. However, to establish whether this effect was specific to the type of cognitive processing targeted by the digit sequence working memory manipulation, or whether instead the same results would be found for perceptual processing, Experiment 3 manipulated perceptual processing resources during the same action understanding task.

## Experiment 3

Experiment 3 sought to manipulate perceptual processing resources by combining the action understanding task from Experiment 1 with a visual working memory task to determine whether action identification and/or intention identification are reduced under limited perceptual processing capacity.

If either action identification or intention identification requires the same perceptual processing resources that are used to hold material in visual working memory, then performance on the respective condition should be disrupted under high perceptual load. In contrast, if performance on the action identification and/or the intention identification conditions does not differ as a function of perceptual load, this would indicate that these conditions do not utilise the same perceptual processes that occur within visual working memory.

### Method

#### Participants

A required sample size of 34 participants was calculated based on the same power analysis as in Experiment 2. Hence, 34 native English speakers were recruited via King’s College London recruitment systems and were compensated course credits or a small fee for their time. However, one participant was excluded as their accuracy score on the action understanding task was not significantly different from chance. This resulted in 33 participants (4 males, 3 left-handed) aged 18–45 years (*M* = 21.9, *SD* = 4.95).

#### Stimuli

The stimuli for the visual working memory task were based on those used by Mecklinger and Pfeifer (1996), comprising two-dimensional pattern arrays on a 40 × 40 square grid. In the low-load condition, one element was presented on the array, while in the high-load condition, there were five elements. The elements were arranged so that two elements were not presented in the same row or column of the grid; the horizontal or vertical distance between two elements was at least 8 cells; and the grid was subdivided into 16 segments, in which, for the low-load stimuli, a different segment was used for each trial, whereas for the high-load stimuli, each segment was used at least once but not more than twice across all trials. To produce the mismatching stimuli, one element was moved seven cells: a combination of horizontally and vertically. This adhered to the following rules: the frequency of the direction of movement occurred an equal number of times; and, in the high-load condition, each element position was moved an equal number of times. This ensured that no priming occurred for element location or direction.

Sixteen stimuli were created for each working memory sub-condition—low-load matching, low-load mismatching, high-load matching, and high-load mismatching. Each stimulus thus had a corresponding mismatching version. Each element appeared white on a black background, to ensure that there were minimal retinal after-effects. The same condition-task structure as in Experiment 2 was used to ensure equal combination of action understanding and working memory conditions. Fully factorial combination of all action understanding stimuli and conditions with all working memory sub-conditions resulted in 256 trials, which were divided between four blocks of 64 trials per block, such that each block contained 16 trials per cell of the design (low-load action; low-load intention; high-load action; high-load intention), equally divided between matching and mismatching trials for both the action understanding and working memory tasks. As in Experiment 2, due to fatigue and time constraints, each participant completed two blocks of trials. These were systematically allocated across participants.

The action understanding task was identical to that used in Experiments 1 and 2.

#### Procedure

The procedure was identical to that of Experiment 2, with the exception that the visual, rather than the numerical, working memory stimuli were presented before and after the action understanding task on each trial (see Figure 1c). These stimuli were presented for 1,800 ms, based on the timings used by Mecklinger and Pfeifer (1996). Participants were additionally instructed to pay close attention to all elements in the array.

### Results

The RT data were filtered in the same way as Experiment 2 and inverse efficiency scores were calculated for each condition (see Figure 2).

The inverse efficiency scores for the action understanding task were subjected to repeated measures ANOVA with within-subjects factors of perceptual load (low-load, high-load) and action understanding condition (action, intention). There were no significant main effects of perceptual load or action understanding condition, or interaction between the two.

A repeated measures ANOVA was also conducted on the inverse efficiency scores for the working memory task (see Table 2). There was a significant main effect of load, *F*(1,32) = 61.17, *p* < .001, $\eta_{p}^{2}=65.7\%$ . Participants performed significantly worse during the high-load (*M* = 1,375.6, *SD* = 395.9) than the low-load condition (*M* = 1,031.7, *SD* = 254.6). There was no main effect of action understanding condition and no interaction between load and condition.

#### Exploratory analysis

Although the low-load condition for both cognitive and perceptual load types was intended to be equivalent to a no-load condition (Ahmed & De Fockert, 2012; Fockert et al., 2001; Spunt & Lieberman, 2013), Figure 2 indicates that—at least numerically—participants appeared to perform worse on the low-load conditions of Experiments 2 and 3 than in Experiment 1 where they were not subjected to any load at all. It is possible, therefore, that even low levels of cognitive and/or perceptual load were sufficient to disrupt performance on the action understanding task, compared with a no-load condition. The low-load data from Experiments 2 and 3, and the no-load data from Experiment 1 were therefore combined and subjected to a mixed ANOVA with between-subjects factor of load type (none, low cognitive load, low perceptual load) and within-subjects factor of condition (action identification, intention identification). As this analysis was exploratory and utilises data already reported in Experiments 1–3, all *p* values below are Bonferroni corrected.

There was a significant effect of load type on inverse efficiency scores for action identification, *F*(2,95) = 16.68, *p* < .001, $\eta_{p}^{2}=26.0\%$ . Simple effects analyses revealed that participants’ action identification performance was significantly worse when the task was combined with a low cognitive load (*M* = 1,195.1, *SD* = 298.4) than when the task was completed on its own (*M* = 869.2, *SD* = 161.3), *t*(63) = 5.40, *p* < .001, *d* = 1.36. Similarly, participants’ action identification performance was significantly worse when the task was combined with a low perceptual load (*M* = 1,115.8, *SD* = 219.9) than when completed on its own, *t*(62) = 5.09, *p* < .001, *d* = 1.28. There was no difference in performance in the action identification condition when the task was combined with a low cognitive, compared with a low perceptual, load. Similarly, there was a significant effect of load type on inverse efficiency scores for intention identification, *F*(2,95) = 12.23, *p* < .001, $\eta_{p}^{2}=20.5\%$ . Participants performed significantly worse in the intention identification condition when the task was combined with a low cognitive load (*M* = 1,180.4, *SD* = 287.2), and also when it was combined with a low perceptual load (*M* = 1,108.2, *SD* = 203.1), compared with when the task was completed on its own (*M* = 900.1, *SD* = 202.2); *t*(63) = 4.51, *p* < .001, *d* = 1.13, and *t*(62) = 4.11, *p* < .001, *d* = 1.03, respectively. There was no difference in performance in the intention identification condition when the task was combined with a low cognitive, compared with a low perceptual, load.

### Discussion

The aim of Experiment 3 was to determine whether action and intention identification require perceptual processing resources. We found no difference in performance for either action identification or intention identification between the low-load and high-load conditions. However, the comparison of both low-load cognitive and perceptual processing conditions to baseline conditions from Experiment 1 demonstrates that performance was significantly disrupted for action identification and intention identification for both cognitive and perceptual low-load conditions. Therefore, reducing the availability of perceptual processing to a small extent impacts on action and intention identification, but further loading of perceptual processing does not impact further on these processes.

One possibility for the lack of further disruption in the high-load perceptual processing condition could be that the high-load condition was less demanding than anticipated. However, participants performed significantly worse in the high-load than low-load condition of the working memory task, indicating that further significant disruption to perceptual capacity occurred.

In sum, Experiment 3 found that reducing perceptual processing capacity impairs action and intention identification compared with baseline but reducing perceptual processing further does not additionally impact on these processes.

## General discussion

The first aim of the present study was to design a new action understanding task which targets two action understanding processes: action identification and intention identification. The results of Experiment 1 indicate that after controlling for a range of variables previously confounding other action understanding tasks, a significant difference in performance remained between the two action understanding conditions. This suggests that the task used in Experiment 1 is measuring two distinct action understanding processes and can be used in a range of experimental designs to investigate these processes further.

The second aim of the study was to determine whether action identification and intention identification are automatic or involve inferential processing, specifically cognitive and/or perceptual processing resources. Experiment 2 combined the action understanding task with a numerical working memory task in a dual-task design to investigate whether action and intention identification could occur under limited cognitive processing capacity. The results from Experiment 2, when compared with those of Experiment 1, indicated that both action and intention identification were impaired under low cognitive load and were further disrupted by additional cognitive load, implying that both action identification and intention identification utilise cognitive processing resources.

Similarly, Experiment 3 combined the action understanding task with a visual working memory task to determine whether action and intention identification are impaired under limited perceptual processing capacity. When compared with Experiment 1, both action and intention identification were impaired under low perceptual load, but these processes were not further disrupted by additional perceptual load, implying that both processes require non-automatic perceptual processing to be completed efficiently.

Altogether, the results indicate that action identification and intention identification do not take place automatically. Instead, impairment to these processes under low perceptual and cognitive load indicates that both action identification and intention identification draw on controlled processing resources. Moreover, the additional impairment produced for both of these processes under high cognitive load, indicates that both action and intention identification require substantial cognitive processing resources.

One interpretation for the impaired performance on action identification and intention identification under low perceptual load compared with baseline, but not between low and high perceptual load, could be that these processes require central (attentional) resources but not perceptual processing resources. Dual-task experiments measure automaticity by impacting on both central attention (Allen et al., 2006; Baddeley et al., 2009) and domain-specific processing resources, by placing additional demands on both the central executive (Baddeley, 1986) and a specific sub-component of working memory (the phonological loop or visuospatial sketchpad; Baddeley, 2000; Baddeley & Hitch, 1974). While the processing resources involved in the two subcomponents of working memory are thought to be independent (e.g., Baddeley, 2012; Cocchihi et al., 2002) the central executive allocates controlled processing resources across these subcomponents. Therefore, dual-tasks based on working memory may impact on the processing capacity of one of the subcomponents of working memory, but also on attentional resources allocated across subcomponents (Cowan & Morey, 2007).

The low-load working memory conditions in the present study, therefore, could be depleting general attentional resources rather than specific processing resources, due to the limited amount of perceptual or cognitive information required to be held in working memory. As the central executive capacity is limited (Baddeley & Logie, 1999), the low-load conditions may occupy all attentional resources, with no additional depletion of attention within the high-load conditions. Instead, the high-load conditions may deplete the specific processing resources of that particular sub-component, indicating which type of processing resources are required in the task under investigation. Based on this logic, the results of this study suggest that the reduced performance for both action identification and intention identification in the low cognitive and perceptual load conditions indicate the involvement of general attentional resources in these processes. The additional impairment under high cognitive load may indicate that cognitive processing resources are also required for these processes. However, as dual-task designs target both central (attentional) and domain-specific processing resources, the precise distinction between general and domain-specific controlled processing resources cannot be revealed from these data. Further research, specifically targeting these different aspects of controlled processing, is needed to determine whether the impairment found under low perceptual load is due to depletion of central (attentional) or perceptual processing resources.

An alternative explanation for the impairment in performance for both action and intention identification for both low cognitive and low perceptual load could be that holding any type of information within working memory slows down performance compared with completing the task alone, due to non-specific task effects, such as motivation or confusion over task instructions. However, in a dual-task study of imitation conducted by Ramsey et al. (2019), performance on an imitation task was not significantly impaired by holding verbal, visual, or action information in working memory, suggesting that dual-task designs do not necessarily entail a general reduction in performance, and that it is in principle possible to use such designs to uncover evidence of automaticity. Moreover, the current findings were not due to lack of depletion of processing resources by the working memory tasks, as significant impairments in performance on these tasks were found between the low- and high-load conditions. Therefore, the results of the present study are unlikely to be due to non-specific effects of including an additional task, such as motivation, and instead are more likely to be due to the impact of the secondary tasks on attentional and processing resources, demonstrating the requirement of these resources in action and intention identification.

Although neither action identification nor intention identification was found to occur without the requirement of controlled processing resources, it should be noted that if this had been found to be the case, these findings alone would not be sufficient to claim that action identification and intention identification are automatic processes. Instead, these findings would indicate that action and intention identification consist of one automatic aspect, in regard to being efficient, and persisting under concurrent processing load, but further research would be needed to investigate their relationship to other aspects of automatic processing. Automaticity is thought to be a multidimensional construct (Bargh, 1994; Moors & De Houwer, 2006), and it has been argued that for social behaviours to be automatic they must fulfil three requirements: they must be unintentional, not requiring instruction; be stimulus-driven and resistant to top-down control; and be efficient, persisting under concurrent processing load (as discussed in Ramsey et al., 2019). To determine whether action identification and intention identification contain other automatic aspects to that investigated in the present study, future research should explore their intentionality and resistance to top-down control.

One limitation of using dual-tasks to disrupt attentional resources is that often there is a reduction of task costs with practice (e.g., Hazeltine et al., 2002; Oberauer & Bialkova, 2011; Strobach et al., 2012). High dual-task costs at the beginning of practice are typically explained by interference of one or both component tasks owing to a capacity limitation in the cognitive system (e.g., Logan & Gordon, 2001; Pashler, 1994). It is assumed that this interference is reduced with practice, explaining the reduction of dual-task costs over time. While in the present study, participants were not subjected to extensive practice before completing the tasks, it is possible that after a few trials of completing the tasks concurrently, rather than individually, participants were able to learn strategies to complete the tasks, simultaneously resulting in controlled processes not being utilised in the expected way. However, the significant reduction in performance in the high-load condition compared with the low-load condition in each of the dual working memory tasks indicates that the high-load conditions were taxing performance throughout each experiment.

Another limitation of the dual-task design is evident in the present study: performance in the low-load conditions of the action understanding task in both Experiments 2 and 3 was significantly impaired compared with completing the task alone. Therefore, using a low-load condition as a measure of available cognitive or perceptual capacity (Ahmed & De Fockert, 2012; Fockert et al., 2001; Spunt & Lieberman, 2013) may not provide a true baseline measure of performance.

In conclusion, this study presented a novel action understanding task targeting action identification and intention identification and used this task to investigate whether these processes are automatic or instead require cognitive and/or perceptual processing resources. The results suggest that both action identification and intention identification require cognitive and perceptual processing resources, contradicting direct accounts of action understanding (Gallagher, 2008; Rizzolatti & Sinigaglia, 2007). Instead, the findings support dual-stage accounts of action understanding (Cole et al., 2019; de Lange et al., 2008; Hamilton & Marsh, 2013; Libero et al., 2014; Spunt & Lieberman, 2012; Spunt et al., 2011), in which the processing carried out by sensorimotor brain areas, such as those containing mirror neurons, is combined with inferential processing resources to complete action and intention identification.

## Supplemental Material

sj-docx-1-qjp-10.1177_17470218221078019 – Supplemental material for Is action understanding an automatic process? Both cognitive and perceptual processing are required for the identification of actions and intentions Click here for additional data file. Supplemental material, sj-docx-1-qjp-10.1177_17470218221078019 for Is action understanding an automatic process? Both cognitive and perceptual processing are required for the identification of actions and intentions by Emma L Thompson, Emily L Long, Geoffrey Bird and Caroline Catmur in Quarterly Journal of Experimental Psychology
