# Supplementary material for: Is action understanding an automatic process? Both cognitive and perceptual processing are required for the identification of actions and intentions
Source: Q J Exp Psychol (Hove). 2022 Feb 18;76(1):70–83. doi: 10.1177/17470218221078019 (PMC9773155; doi:10.1177/17470218221078019)
Supplement: sj-docx-1-qjp-10.1177_17470218221078019 – Supplemental material for Is action understanding an automatic process? Both cognitive and perceptual processing are required for the identification of actions and intentions [file sj-docx-1-qjp-10.1177_17470218221078019.docx]

**Supplementary Material for:**

Is action understanding an automatic process? Both cognitive and perceptual processing are required for the identification of actions and intentions

Emma L. Thompson^a,b^, Emily L. Long^c^, Geoffrey Bird^c,d^ and Caroline Catmur^a^

**Piloting**

**Pilots 1 and 2: validating word/image pairings**

**Pilot 1.** Four volunteers participated in a pilot study in order to validate the word / image pairings of twenty initially selected stimuli. Participants completed one block of the action understanding task, as detailed in the manuscript. Trials were presented in a random order. Four additional tasks were then completed to collect ratings on the suitability of the stimuli and word phrases. In task one, participants were presented with each image, and were asked to freely generate the action and intention they thought was depicted in the image. Task two consisted of each word phrase presented to participants in a randomized order and participants were asked to indicate whether the word was an action or intention based on a scale of 1 = “definitely action”, 2 = “probably action”, 3 = “probably intention” and 4 = “definitely intention”. In task three, participants were asked to select, from lists containing all of the action and intention word phrases, one action word phrase and one intention word phrase that best represented each image. Finally, in task four, each image was presented alongside its corresponding action and intention phrases and participants were asked to rate how well they thought each phrase described the image on a scale of: 1 = “does not describe picture at all”; 2 = “describes picture poorly”; 3 = “somewhat describes picture”; 4 = “describes picture well”; 5 = “describes picture very well”. The stimuli with the highest ratings from task four were selected and those with poorer ratings (values < 3) were altered.

**Pilot 2.** The new stimuli were then subjected to another round of piloting. Nine additional participants took part in the second pilot, in which only tasks two and four were administered. Tasks one and three were initially used to determine whether participants preferred any alternative word phrases to those used for each image, and as such were not deemed necessary for this round of piloting.

**Pilots 1 and 2 combined.** Across the two rounds of piloting, the responses from tasks two and four were combined, resulting in data from 13 participants (four males, 12 right-handed) aged 18 - 26 years (Mean = 20.69, standard deviation (SD) = 2.21). From these responses, 16 stimuli were selected as the final set of stimuli for the action understanding task (see Table 1 in the main manuscript).

**Pilot 3: determining effect size**

**Participants and Procedure.** Thirty-two native English speakers were recruited via King’s College London recruitment email and took part in the pilot study. One participant was removed due to failure to follow instructions correctly. This resulted in 31 participants (seven males, five left-handed) aged 18 - 40 years (Mean = 22.06, SD = 5.58). All participants were compensated a small fee or course credits for their time. The task and procedure was identical to that described in the main manuscript (Experiment 1).

**Results.** Paired-sample t-tests showed that participants were significantly faster to respond to the action condition (Mean = 870.0ms, SD = 161.3) than the intention condition (Mean = 900.9ms, SD = 202.2), *t*(30) = 2.24, *p* = .033, *d* = 0.47; but there was no significant difference in accuracy between the action (Mean = 79.5%, SD = 7.22) and intention conditions (Mean = 80.2%, SD = 8.06), *t*(30) = 0.64, *p* = .529.

**Experiment 1**

**Supplementary Results**

**Response time (RT) and accuracy data.** Figure S1 presents the RT data, and Figure S2 the accuracy data, for Experiments 1, 2 and 3.


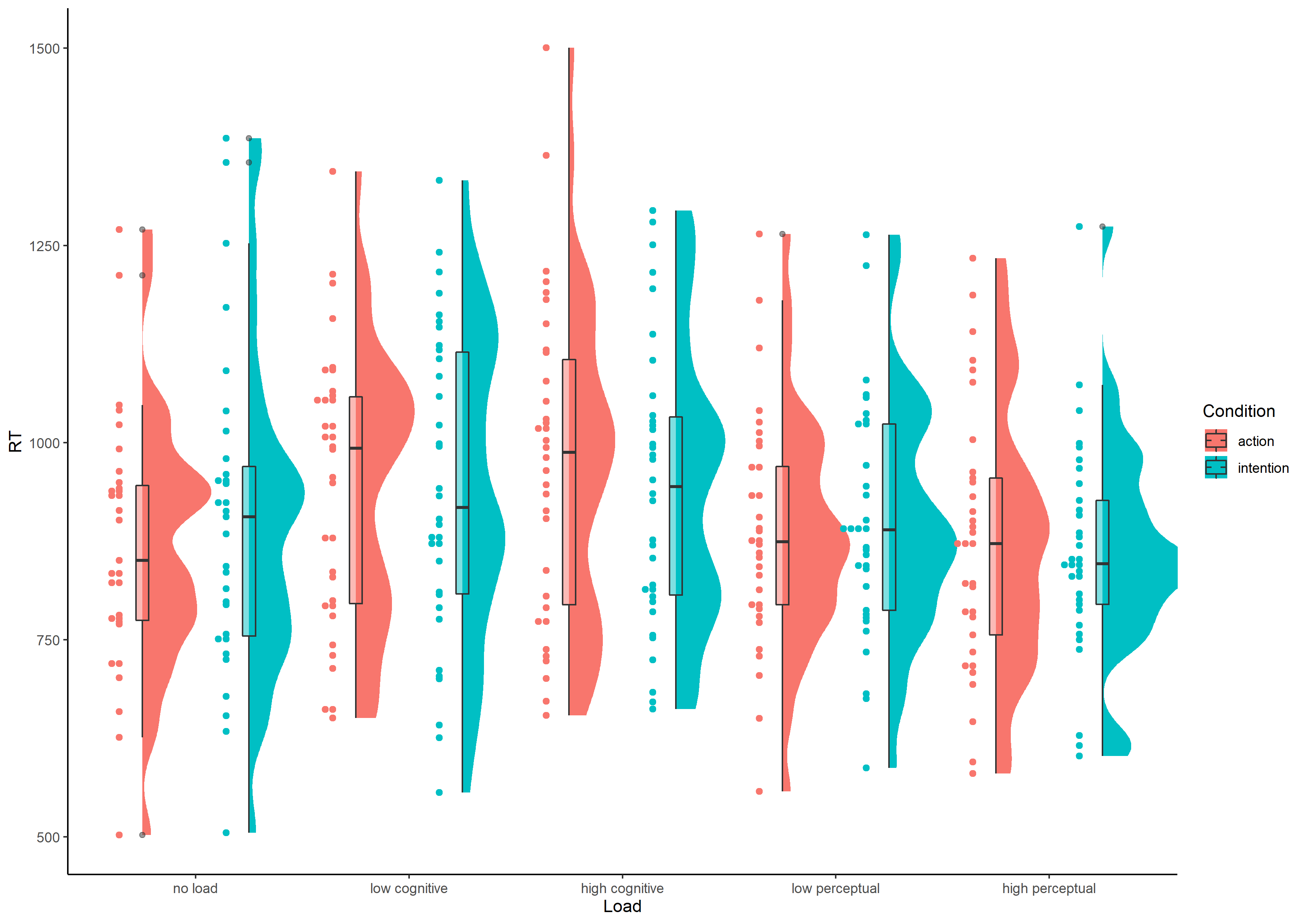


**Figure S1.** Response times (RT) for each load and action understanding condition across the three experiments (Experiment 1: No Load; Experiment 2: Cognitive Load; Experiment 3: Perceptual Load).


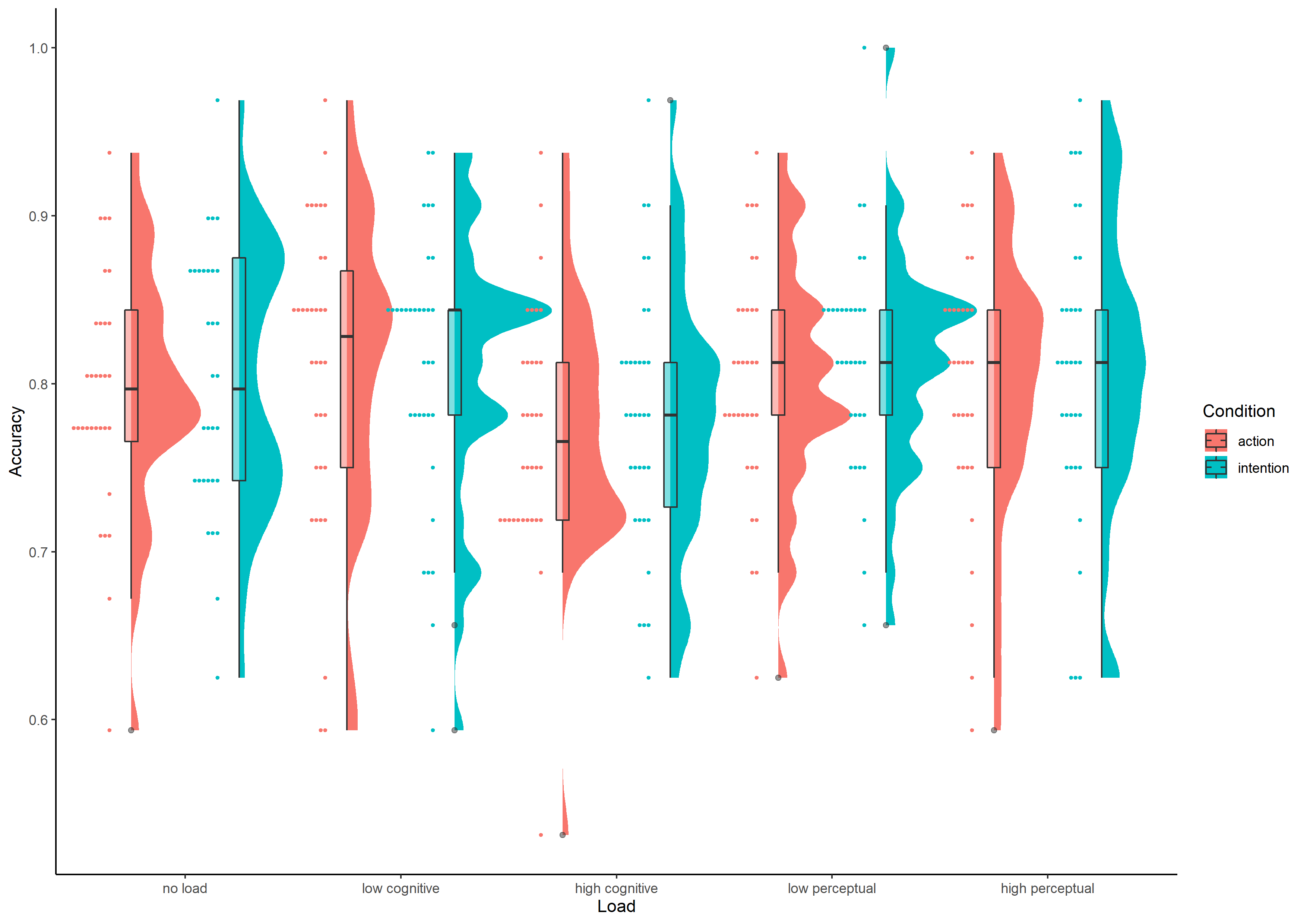


**Figure S2.** Accuracy (proportion correct) for each load and action understanding condition across the three experiments (Experiment 1: No Load; Experiment 2: Cognitive Load; Experiment 3: Perceptual Load).

**Multilevel modelling results.** RT and accuracy data were analysed using multi-level linear mixed models. Modelling was performed using the lme4 package (Bates, Mächler, Bolker, & Walker, 2014) for R (R Core Team, 2020). The fitted models included stimulus image and participant as random effects, and condition as a fixed effect (full equations are given below). Consistent with the inverse efficiency analysis, response times were significantly longer for the intention condition compared to the action condition, *B* = 29.97, *SE* = 10.55, *t* (3053) = 2.84, *p* = .005. No effect of condition was detected in the accuracy data (*p* = .563).

**Multilevel model equations.**

RT: Response Time ~ Condition + (1 | Participant) + (1 | Image)

Acc: Accuracy ~ Condition + (1 | Participant) + (1 | Image)

*Note.* RT = reaction time model, Acc = accuracy model. Due to the binary nature of the accuracy variable (i.e., correct/incorrect), a binomial generalised linear mixed model was fitted (thus using the glmer function with family = binomial), whilst the continuous response time variable merited the use of the lmer function.

**Experiment 2**

**Supplementary Results**

**Inverse efficiency analysis.** Tables S1 and S2 present the full statistics for the analyses of the inverse efficiency data for the action understanding (S1) and working memory (S2) tasks.

| Effect | df | *F* | *p* | *η^2^_p_* |
| --- | --- | --- | --- | --- |
| Cognitive Load | 1,33 | 11.26 | .002 | 25.4% |
| Action Understanding Condition | 1,33 | 2.63 | .115 | 7.4% |
| Load x Condition | 1,33 | 1.00 | .326 | 2.9% |

**Table S1.** Statistics from the analysis of the inverse efficiency scores for the action understanding task in Experiment 2.

| Effect | df | *F* | *p* | *η^2^_p_* |
| --- | --- | --- | --- | --- |
| Cognitive Load | 1,33 | 220.19 | <.001 | 87.0% |
| Action Understanding Condition | 1,33 | 2.51 | .123 | 7.1% |
| Load x Condition | 1,33 | 0.91 | .348 | 2.7% |

**Table S2.** Statistics from the analysis of the inverse efficiency scores for the working memory task in Experiment 2.

**Multilevel modelling results.** RT and accuracy data were analysed using multi-level linear mixed models including stimulus image and participant as random effects, and condition and cognitive load as fixed effects (full model equations are given below). Consistent with the inverse efficiency analysis, high cognitive load resulted in more errors (lower accuracy) than low cognitive load, *B* = 0.24, *SE* = 0.11, *z* = 2.20, *p* = .028. There was no effect of condition in the accuracy data, *p* = .627. Response times were, however, longer for action identification than for intention identification, *B* = -28.53, *SE* = 13.85, *t* (3328) = -2.06, *p* = .040. There was no effect of cognitive load on reaction times, *p* = .090. No interaction between condition and cognitive load was observed in either accuracy or response times, *p*s > .235.

**Multilevel model equations.**

RT: Response Time ~ Condition * Load + (1 | Participant) + (1 | Image)

Acc: Accuracy ~ Condition * Load + (1 | Participant) + (1 | Image)

*Note.* RT = reaction time model, Acc = accuracy model. Due to the binary nature of the accuracy variable (i.e., correct/incorrect), a binomial generalised linear mixed model was fitted (thus using the glmer function with family = binomial), whilst the continuous response time variable merited the use of the lmer function.

**Experiment 3**

**Supplementary Results**

**Inverse efficiency analysis.** Tables S3 and S4 present the full statistics for the analyses of the inverse efficiency data for the action understanding (S3) and working memory (S4) tasks.

| Effect | df | *F* | *p* | *η^2^_p_* |
| --- | --- | --- | --- | --- |
| Perceptual Load | 1,32 | 0.56 | .458 | 1.7% |
| Action Understanding Condition | 1,32 | 0.62 | .436 | 1.9% |
| Load x Condition | 1,32 | 0.19 | .664 | 0.6% |

**Table S3.** Statistics from the analysis of the inverse efficiency scores for the action understanding task in Experiment 3.

| Effect | df | *F* | *p* | *η^2^_p_* |
| --- | --- | --- | --- | --- |
| Perceptual Load | 1,32 | 61.17 | <.001 | 65.7% |
| Action Understanding Condition | 1,32 | 0.62 | .438 | 1.9% |
| Load x Condition | 1,32 | 0.03 | .871 | 0.1% |

**Table S4.** Statistics from the analysis of the inverse efficiency scores for the working memory task in Experiment 3.

**Multilevel modelling results.** RT and accuracy data were analysed using multi-level linear mixed models including stimulus image and participant as random effects, and condition and perceptual load as fixed effects. Consistent with the inverse efficiency analysis, no main effects of condition or perceptual load were found for either RT or accuracy data and no interactions were observed, *p*s > .243.

**Multilevel model equations.**

RT: Response Time ~ Condition * Load + (1 | Participant) + (1 | Image)

Acc: Accuracy ~ Condition * Load + (1 | Participant) + (1 | Image)

*Note.* RT = reaction time model, Acc = accuracy model. Due to the binary nature of the accuracy variable (i.e., correct/incorrect), a binomial generalised linear mixed model was fitted (thus using the glmer function with family = binomial), whilst the continuous response time variable merited the use of the lmer function.

**Supplementary References**

Bates, D., Mächler, M., Bolker, B., & Walker, S. (2014). Fitting linear mixed-effects models using lme4. *arXiv preprint arXiv:1406.5823*.

R Core Team (2020). R: A language and environment for statistical computing. R Foundation for Statistical Computing, Vienna, Austria
